# Adaptive Path Selection for Link Loss Inference in Network Tomography Applications

**DOI:** 10.1371/journal.pone.0163706

**Published:** 2016-10-04

**Authors:** Yan Qiao, Jun Jiao, Yuan Rao, Huimin Ma

**Affiliations:** 1 School of Information and Computer Science, Anhui Agricultural University, Hefei, Anhui, China; 2 State Key Laboratory of Networking and Switching Technology, Beijing University of Posts and Telecommunications, Beijing, China; IUMPA - Universitat Politecnica de Valencia, SPAIN

## Abstract

In this study, we address the problem of selecting the optimal end-to-end paths for link loss inference in order to improve the performance of network tomography applications, which infer the link loss rates from the path loss rates. Measuring the path loss rates using end-to-end probing packets may incur additional traffic overheads for networks, so it is important to select the minimum path set carefully while maximizing their performance. The usual approach is to select the maximum independent paths from the candidates simultaneously, while the other paths can be replaced by linear combinations of them. However, this approach ignores the fact that many paths always exist that do not lose any packets, and thus it is easy to determine that all of the links of these paths also have 0 loss rates. Not considering these *good* paths will inevitably lead to inefficiency and high probing costs. Thus, we propose an adaptive path selection method that selects paths sequentially based on the loss rates of previously selected paths. We also propose a theorem as well as a graph construction and decomposition approach to efficiently find the most valuable path during each round of selection. Our new method significantly outperforms the classical path selection method based on simulations in terms of the probing cost, number of accurate links determined, and the running speed.

## Introduction

The robustness of communication networks is extremely important for both users and network service providers. However, as the network grows in terms of size and diversity, it becomes increasingly difficult to monitor the characteristics of the network interior, such as the link loss rates and packet latency. The main problems are as follows [1]: i) general organizations have administrative access to only a small fraction of the network’s internal nodes, whereas commercial factors often prevent organizations from sharing internal performance data; and ii) the servers and routers in the network are usually operated by businesses, which may be unwilling or unable to cooperate with the collection of network traffic measurements for network management. Thus, monitoring the network interior must rely on end-to-end measurements (e.g., paths in overlay networks).

Network performance tomography (or network tomography) is a powerful tool for inferring the performance characteristics of the network interior by correlating sets of end-to-end measurements [1][2][3]. This method formulates the problem of inferring link characteristics from end-to-end path measurements as a large linear system. The link metrics can then be calculated by solving the linear equations in the system. The end-to-end measurements probes injected into the network may incur additional traffic overheads, so it is important to select the paths carefully so the desired inference capability can be achieved with as few probes as possible.

Given a set of candidate paths between monitors, the state-of-the-art solutions select a subset of the candidates to maximize the probing information. Most previous methods select the maximum set of independent paths by finding an arbitrary basis of the linear system [2][4] because the remaining paths can be represented linearly based on them. In other methods, the minimum paths are selected that can determine all of the identifiable links (see the “Definitions and Problem Formulation” section for details) as well as covering all of the unidentifiable links [3]. In addition, Ma et al. [5] tried to construct the optimal path set with the maximum capacity to identify the network links in an environment with controllable routing. However, all previously described methods select the probing paths simultaneously and then infer the link characteristics by using all of the probing results. In this study, we argue that the selection, probing, and inference processes should be conducted alternately, thereby dramatically reducing both the overall number of probing paths required and the computational time needed to select the paths, as well as accurately determining many more network links.

The method proposed in this study is motivated by two observations. i) A considerable proportion of the end-to-end paths appear to have *good* states (e.g., loss rates of nearly 0 or 0 latency) in general communication networks. The *good* states of paths indicate that all of the links on them also have *good* states, so we can easily determine the characteristics of these links (e.g., loss rates of 0 or 0 latency) from the *good* paths. ii) Some links in the network play more important role than others, and thus observing them in advance can greatly facilitate the determination of the remaining links in the network. Similar to the problem of untangling a mess of kinked cables, if we cut one of the key cables, the others can be disengaged accordingly. In summary, if we can obtain some of the metrics for the links from the *good* paths in advance, the determining the remaining links may be much easier.

In this study, we propose a novel adaptive path selection mechanism for network tomography. Instead of selecting, probing, and inferring simultaneously, we conduct the three procedures alternately in order to make use of the probing results obtained from previously selected paths to aid the selection process. In particular, we execute the following procedures repeatedly: i) select the path for observation that provides the most help for determining the current linear system; ii) probe the path once it has been selected; and iii) remove links that can be determined from the current probing results, before returning to i) in the next round. This study makes three main contributions as follows.

1. We state and prove Theorem 2 based on Theorem 1 from our previous study [6] to define the types of links that are more important than others in the inference problem. We also design an efficient approach for identifying the most important links using the graph construction and decomposition method.
2. We develop a path selection method for network tomography to sequentially select paths from the candidates. This method has two steps. The first step is selecting the minimum path set that can cover all network links. All of the paths and links form an original linear system. The second step is selecting the paths that are most helpful for solving the linear system based on Theorem 2 and the graph construction and decomposition method. The number of final paths selected is even smaller than the rank of the linear system.
3. We confirmed the benefits of our proposed method compared with previous approaches using realistic network scenarios based on simulations. All of the results strongly indicated that our new method significantly improved the network tomography performance for applications in terms of the probe cost, number of links determined, and the running speed. In particular, when the networks contained less than 10% lossy links, our method only required half of the previous method’s probing costs to accurately determine even more links than all of the candidate paths.

The remainder of this paper is organized as follows. First, we survey related research in the “Related Work” section. Next, we present the definitions and formally describe our problem in the “Definitions and Problem Formulation” section. In the “Observations” section, we consider some characteristics of the paths and links in network tomography applications, and we then propose some fundamental concepts motivated by the observed characteristics in the “Fundamentals of Path Selection” section. We present our path selection algorithm in the “Adaptive Path Selection For Loss Inference” section. Finally, we evaluate our new method based on realistic topologies in the “Evaluation” section, before giving our concluding remarks in the “Conclusion” section.

## Related Work

Network tomography techniques have been proposed to acquire internal network states by probing the end-to-end paths among monitors located at the network edges instead of monitoring every network element directly. These methods have been widely used (although they are not limited to these areas) in the fields of individual link characteristic inference [3], network topology inference [7], and estimating the complete set of end-to-end measurements from an incomplete set [8]. In this study, we consider the inference of link characteristics based on end-to-end measurements. There are two main problems with this type of network tomography application: selecting a set of minimum paths to reduce the traffic overheads while maximizing the performance, and accurately inferring all of the link characteristics using the probing results obtained from the selected paths.

The first problem was addressed by [2][3][5] and [9]. Chen et al. [2] first proposed the selection of independent paths by finding the basis of the linear system through QR decomposition with column pivoting [10]. The measurements for the remaining paths can be inferred from the selected paths. Ma et al. [5] proposed a spanning tree-based path construction method to construct linearly independent monitor-to-monitor paths with a complexity of *O*(*mn*) for use in an environment where all of the network routers support the source routing policy. Zheng et al. [3] selected a minimum path set that can identify all identifiable links as well as cover all unidentifiable links. Tati et al. [9] considered the presence of link failures in current networks and proposed RoMe to tolerate link failures by selecting the path set with the maximum expected rank. All of these approaches simultaneously select paths from the candidates and probe them together to obtain the required metrics on these paths. However, this selection method has the following drawbacks. First, probing all of the selected paths may cause a burst in traffic on the network, which may have a negative impact on both the network performance and the probing results. Second, these methods fail to utilize the important information from *good* paths, thereby inevitably missing a considerable proportion of links that can be determined based on the *good* paths.

Previous studies of the second problem can be broadly classified as algebraic and statistical. The linear system is mostly underdetermined (i.e., innumerable solutions to the link characteristics satisfy the measurements on the paths), but both approaches try to find the solution that is most similar to the actual solution. Algebra-based approaches model the link characteristics as unknown constants and then compute the link characteristics from the path measurements by applying linear algebra techniques [4][11]. Statistics-based approaches model the link characteristics as random variables with given (or learnt) prior probability distributions and they employ statistical techniques to estimate the posterior distributions from single or multiple measurements on paths [12][13][14]. All of these methods infer the link metrics using the probing results obtained on the selected paths after the selection and probing stages. However, the metrics on links cannot remain stable throughout the whole process. Furthermore, executing the selection and inference procedures separately may affect accuracy of the inference results.

In the proposed method, for the first time, we merge the procedures for path selection, path probing, and link metric inference in network tomography applications. Thus, the probes are injected sequentially into the network, thereby avoiding the presence of burst traffic. Moreover, the critical information from *good* paths can be utilized to reduce the number of overall paths that need to be probed, as well as determining more link characteristics.

In the “Evaluation” section, we compared our new algorithm (APSA) with SelectPath [2]. Although several recent methods have been proposed to address the tomographic problems [12][13][14][15][16][9][17], most of them are not comparable with our algorithm. For example, [12][13] and [14] do not select paths before they perform tomography, while our algorithm focuses on the path selection stage; [15][16] and [17] select monitoring paths to detect or locate the failures, but our algorithm aims to determine the loss rates of links; [9] selects paths before the tomography stage under the condition that there are link failures in the network, which is outside the scope of this paper. We choose SelectPath as the baseline not only because it works on problems similar to those of our algorithm but also because it is one of the most representative path selection algorithms that has been widely approved of in the research community.

### Definitions and Problem Formulation

We take the loss rates inference problem as an example to explain our new proposed network tomography approach. The formulation is similar to that of our previous study [1]. Let *G* = (*υ*, *ε*) denote the network with the set of nodes *υ* and links *ε*. The numbers of nodes and links are denoted by |*υ*| and |*ε*|, respectively. We define a path *P*_*i*_ as a sequence of links starting from a source host and ending at a destination host. All of the paths in the network form the path set **P**. The number of paths in **P** is denoted by |**P**|. In general, the nodes at the edge of the network may act as monitors. A single path is assumed between each pair of monitors, which is usually provided by routing algorithms on the Internet [18]. For a given network *G* = (*ν*, *ε*) and a path set **P**, we define the routing matrix **R** with dimension *M* × *N*, where *M* = |**P**| and *N* = |**ε**|, as follows: each row of **R** represents a path in the network and the columns represent links, i.e., **R**_*ij*_ = 1 when path *P*_*i*_ traverses link *e*_*j*_, and **R**_*ij*_ = 0 otherwise.

**Fig 1:**
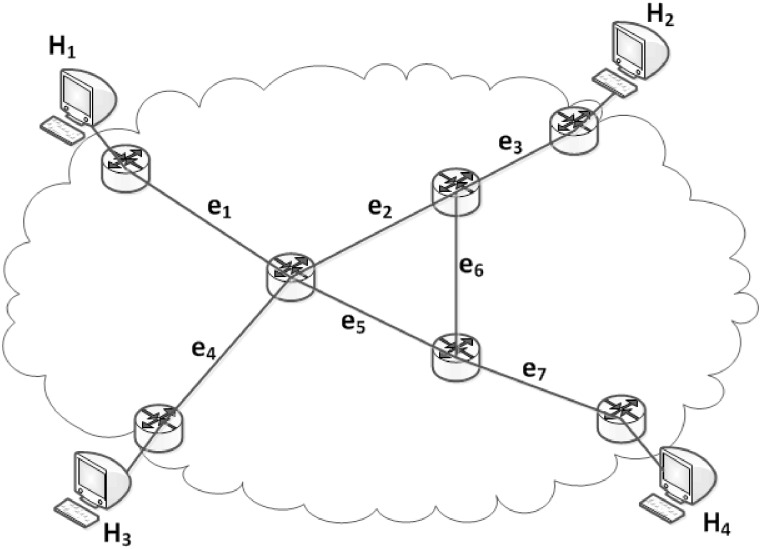
Example of a Network Topology.

For example, Fig 1 shows a network topology with 7 links and 4 monitors (*H*_1_ ∼ *H*_4_), and there are 6 paths among the monitors in the network (as shown in Table 1).

**Table 1:** Set of Paths in Fig 1.

Paths	Routes
*P*_1_: *H*_1_ → *H*_2_	*e*_1_, *e*_2_, *e*_3_
*P*_2_: *H*_1_ → *H*_3_	*e*_1_, *e*_4_
*P*_3_: *H*_1_ → *H*_4_	*e*_1_, *e*_5_, *e*_7_
*P*_4_: *H*_2_ → *H*_3_	*e*_2_, *e*_3_, *e*_4_
*P*_5_: *H*_2_ → *H*_4_	*e*_3_, *e*_6_, *e*_7_
*P*_6_: *H*_3_ → *H*_4_	*e*_4_, *e*_5_, *e*_7_

The routing matrix **R** in Table 1 is as follows.

$$R=\left(\begin{array}{ccccccc} 1 & 1 & 1 & 0 & 0 & 0 & 0\\ 1 & 0 & 0 & 1 & 0 & 0 & 0\\ 1 & 0 & 0 & 0 & 1 & 0 & 1\\ 0 & 1 & 1 & 1 & 0 & 0 & 0\\ 0 & 0 & 1 & 0 & 0 & 1 & 1\\ 0 & 0 & 0 & 1 & 1 & 0 & 1 \end{array}\right)$$ (1)

The rank of **R** is 5, which means that at most 5 row vectors are linear independent. We say that the paths are *independent* if the rows representing them are linear independent. This also means that none of the paths can be replaced by other paths in the *independent* path set. For example, *P*_1_, *P*_2_, *P*_3_, *P*_4_, and *P*_5_ are independent paths, but *P*_6_ is dependent on them because it can be replaced by *P*_3_ − *P*_1_ + *P*_4_ in Eq 1.

Let ${\hat{\phi }}_{i}$ be the random variable given the fraction of a number of probe packets that arrive correctly at the destination monitor in the current measurement. Let ${\hat{\phi }}_{e_{j}}$ be the fraction of packets from all paths passing through link *e*_*j*_ that have not been lost at that link. For any path *P*_*i*_, we define its transmission rate as $\phi_{i}=E\left({\hat{\phi }}_{i}\right)$. Similarly, the transmission rate of link *e*_*j*_ can be defined as $\phi_{e_{j}}=E\left({\hat{\phi }}_{e_{j}}\right)$.

Given the routing matrix **R**, the relationship between the transmission rates of paths in **P** and the transmission rate of links in *ε* can be formulated as follows.

$$\begin{array}{c} \phi_{i}=\prod_{j=1}^{N}\phi_{e_{j}}^{\left(R_{ij}\right)} \end{array}$$ (2)

Taking the logarithms on both sides of Eq(2), we can rewrite this equation as,

$$\begin{array}{c} \log\phi_{i}=\sum_{j=1}^{N}R_{ij}\log\phi_{e_{j}}. \end{array}$$ (3)

Let *X*_*j*_ = log *ϕ*_*e*_*j*__ and *Y*_*i*_ = log *ϕ*_*i*_, which are grouped in vector **X** = {*X*_1_, ⋯, *X*_*N*_} and **Y** = {*Y*_1_, ⋯, *Y*_*M*_}, respectively. Then, Eq (3) is equivalent to

$$\begin{array}{c} RX=Y \end{array}$$ (4)

To identify the loss rates (loss rate = 1− transmission rate) of individual links, it is necessary to solve the linear equations Eq (4). Normally, the number of rows in **R** is much larger than the number of columns. Unfortunately, in most cases, **R** is still column-deficient. Nevertheless, partial links in the network can be uniquely determined by the measurement results, which we call the *identifiable* links and the remaining links are the *unidentifiable* links.

For example, in Fig 1, links *e*_1_ and *e*_4_ are identifiable links. The loss rates of paths *P*_1_, *P*_2_, and *P*_4_ have been obtained, so we can construct the following equations:

$$\begin{array}{c} \left\{\begin{array}{ccc} & X_{1}+X_{2}+X_{3} & =Y_{1}\\ & X_{1}+X_{4} & =Y_{2}\\ & X_{2}+X_{3}+X_{4} & =Y_{4} \end{array},\right. \end{array}$$ (5)

where *Y*_*i*_ = log *ϕ*_*i*_ and *X*_*j*_ = log *ϕ*_*e*_*j*__. *X*_1_ can be calculated by $\frac{Y_{1}+Y_{2}-Y_{4}}{2}$ and *X*_4_ can be calculated by $\frac{Y_{2}+Y_{4}-Y_{1}}{2}$. However, none of the links *e*_2_, *e*_3_, *e*_5_, *e*_6_, and *e*_7_ can be determined even though all of the six paths have been measured.

In fact, the number of end-to-end paths |**P**| is in the order of *O*(|*υ*|^2^). Thus, probing all of the paths will incur considerable probing time costs as well as large traffic overheads. Therefore, it is necessary to carefully select the probing paths that are most useful for inference. Previous methods either select independent paths (such as *P*_1_, *P*_2_, *P*_3_, *P*_4_, and *P*_5_) or paths that can identify all of the identifiable links (such as *P*_1_, *P*_2_, and *P*_4_). In the present study, our goal is to efficiently select and probe the minimum paths from the candidates that can accurately determine the maximum network links.

## Observations

In this section, we present two observations that can significantly improve the path selection performance of network tomography techniques.

### Observation 1: Help From *Good* Paths

Links that are classified as *unidentifiable* can also be determined accurately. If we observe that the loss rate of a path is 0, then we know that all of the links on this path also have 0 loss rates. Thus, we define paths with near-zero loss rates as *good* paths.

**Fig 2:**
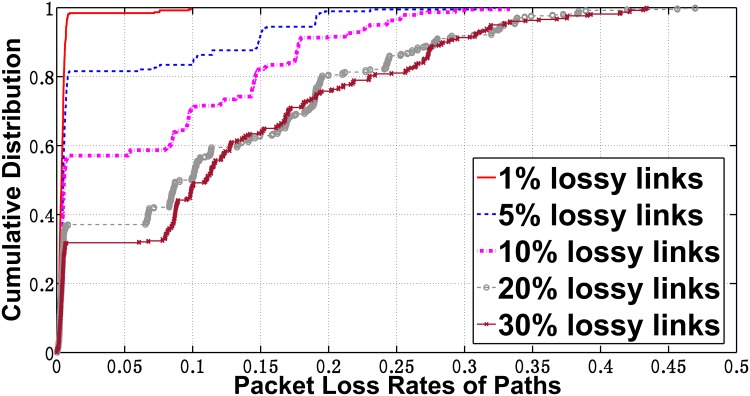
Cumulative Distribution of the Loss Rates on Paths.

The links in good states always comprise the majority of the links in general networks. Thus, a considerable proportion of the paths will have almost 0 loss rates. We show the cumulative distribution of the loss rates on paths under different fractions of lossy links in Fig 2. In our experiments, we used the realistic *AS*1239 topology from the Rocketfuel Project [19]. The detailed settings are provided in the Simulation section. According to Fig 2, over 98% of the paths are *good* paths when 1% of the links lose packets. This proportion reaches 32% even when 30% of the links lose packets. In the remainder of this study, we consider a path as being *good* if the path loss rate is under 2%.

### Observation 2: Important Paths

Some of the *good* paths may play important roles during path selection for network tomography because they contain specific important links. Observing these paths in advance can dramatically reduce the overall probe costs as well as determining more unidentifiable links. For example, as shown in Table 1, if we first probe path *P*_5_ and observe its transmission rate *ϕ*_5_ = 1 (i.e. 0 loss rates), then we can also determine the transmission rates *ϕ*_*e*_3__, *ϕ*_*e*_6__, and *ϕ*_*e*_7__ as 1. After link *e*_*i*_ has been determined as a *good* link, it can be removed directly from the linear system because its corresponding value is *X*_*i*_ = log1 = 0. Next, after removing *e*_3_, *e*_6_, and *e*_7_ from the linear system, we can obtain a new matrix **R**^**′**^.

$$\begin{array}{c} R^{\prime }= \end{array}\left(\begin{array}{cccc} 1 & 1 & 0 & 0\\ 1 & 0 & 1 & 0\\ 1 & 0 & 0 & 1\\ 0 & 1 & 1 & 0\\ 0 & 0 & 1 & 1 \end{array}\right)$$ (6)

Hence, all of the links are identifiable because **R**′ has a full column-rank.

Alternatively, if we first select *P*_2_ and observe *ϕ*_2_ = 1, then after removing *e*_1_ and *e*_4_, the new matrix is as follows.

$$\begin{array}{c} R^{\prime \prime }= \end{array}\left(\begin{array}{ccccc} 1 & 1 & 0 & 0 & 0\\ 0 & 0 & 1 & 0 & 1\\ 1 & 1 & 0 & 0 & 0\\ 0 & 1 & 0 & 1 & 1\\ 0 & 0 & 1 & 0 & 1 \end{array}\right)$$ (7)

In Eq 7, the rank of matrix **R**″ is 3 and none of the links can be determined.

Obviously, *P*_5_ is much more important than *P*_2_ in the example system. In the next section, we state several fundamental concepts related to the sequential determination of the most important path in the current system.

## Fundamentals of Path Selection

Due to the existence of *good* paths, the links that can be determined are not limited to the *identifiable* links. Thus, the general solutions that select paths can only determine all of the identifiable links and they are far from perfect. Therefore, it is natural to question whether a subset of paths exists with the ability to determine as many links as all of the candidate paths, and the answer is yes. Furthermore, it is interesting that the number of paths required is even less than the system rank. In this section, we state and prove several fundamental concepts related to path selection, and we then present a graph construction and decomposition method for identifying the most important path in each round.

### Identifying all of the Identifiable Links in the Linear System

Given the routing matrix **R** where the rows represent all of the candidate paths, the identifiable links in the linear system can be obtained according to Theorem 4.1 from our previous study [6].

Assume that **R** is an *m* by *n* matrix, and the rank of **R** is *r* (let *r* < *n* because all links are identifiable if *r* = *n*). Let *N*(**R**) denote the null space of **R**, *i*.*e*., for any vector *η* ∈ *N*(**R**), **R** *η* = 0. {*η*_1_, *η*_2_, ⋯, *η*_*n* − *r*_} represents an arbitrary basis for *N*(**R**), where *η*_*i*_ = {*α*_*i*1_, *α*_*i*2_, ⋯, *α*_*in*_}, 1 ≤ *i* ≤ *n* − *r*.

**Theorem 1**. *Link e*_*j*_, *which is represented by the j* − *th column of **R***, *can be uniquely identified if and only if for all η*_*i*_, 1 ≤ *i* ≤ *n* − *r*, *α*_*ij*_ = 0.

*Proof*. See [6].

Thus, all of the identifiable links can be found by calculating a basis of the null space of **R**.

### Distinct Roles of Identifiable and Unidentifiable Links

According to Theorem 1, the network links can be divided into two types: identifiable links and unidentifiable links. As mentioned in Observation 2, removing some of the links will make other unidentifiable links become identifiable. In this section, we state Theorem 2 based on Theorem 1 to define the types of links that can help to determine more unidentifiable links.

**Theorem 2**. *The removal of identifiable links will not affect the attributes of other links (i.e., the remaining links will continue to be identifiable/unidentifiable after removing an identifiable link), but the rank of the system will decreases by 1*.

*Proof*. Suppose that the rank of the *m* × *n* routing matrix **R** in the current linear system is *r*, and the numbers of paths and links are *m* and *n*, respectively. Let *η* = {*η*_1_, *η*_2_, ⋯, *η*_*n* − *r*_} represent an arbitrary basis for *N*(**R**), where *η*_*i*_ = {*α*_*i*1_, *α*_*i*2_, ⋯, *α*_*in*_}, 1 ≤ *i* ≤ *n* − *r*.

Assume that *e*_*x*_ is an identifiable link. According to Theorem 1, *α*_*ix*_ = 0, 1 ≤ *i* ≤ *n* − *r*. Let **R**^**′**^ denote the *m* × (*n* − 1) matrix obtained after removing the *x*-th column in **R**, and $\eta^{\prime }=\left\{\eta_{1}^{\prime },\eta_{2}^{\prime },\ldots ,\eta_{n-r}^{\prime }\right\}$ denotes the vector after removing *α*_*ix*_, 1 ≤ *i* ≤ *n* − *r* from {*η*_1_, *η*_2_, ⋯, *η*_*n* − *r*_}. In particular, $\eta_{i}^{\prime }=\left\{\alpha_{i1},\ldots ,\alpha_{i(x-1)},\alpha_{i(x+1)},\ldots ,\alpha_{in}\right\},1\leq i\leq n-r$.

If we can demonstrate that $\eta^{\prime }=\left\{\eta_{1}^{\prime },\eta_{2}^{\prime },\ldots ,\eta_{n-r}^{\prime }\right\}$ is still a basis for the null space of **R**^**′**^, then the attributes of the remaining links in **R**^**′**^ will be the same as those in the original system. Thus, we need to prove the following three claims. First, **R**^**′**^ ⋅ *η*^**′**^ = 0. Second, $\eta_{1}^{\prime },\eta_{2}^{\prime },\ldots ,\eta_{n-r}^{\prime }$ are mutually linear independent. Finally, the rank of the null space of **R**^**′**^ is *n* − *r*.

i) First, we prove that **R**^**′**^ ⋅ *η*^**′**^ = 0.

Since **R** ⋅ *η* = 0, then we have

$$\begin{array}{c} r_{j1}\alpha_{i1}+r_{j2}\alpha_{i2}+\cdots +r_{jn}\alpha_{in}=0, \end{array}$$ (8)

where 1 ≤ *i* ≤ *n* − *r*, 1 ≤ *j* ≤ *m*.

It is known that *α*_*ix*_ = 0, 1 ≤ *i* ≤ *n* − *r*. Thus, it also holds that

$$\begin{array}{c} r_{j1}\alpha_{i1}+\cdots +r_{j(x-1)}\alpha_{i(x-1)}+r_{j(x+1)}\alpha_{i(x+1)}+\cdots +r_{jn}\alpha_{in}=0, \end{array}$$ (9)

and 1 ≤ *i* ≤ *n* − *r*, 1 ≤ *j* ≤ *m*, i.e., **R**^**′**^ ⋅ *η*^**′**^ = 0.

ii) Next, we prove that $\eta_{1}^{\prime },\eta_{2}^{\prime },\ldots ,\eta_{n-r}^{\prime }$ are mutually linear independent.

Suppose that $\eta_{1}^{\prime },\eta_{2}^{\prime },\ldots ,\eta_{n-r}^{\prime }$ are mutually linear dependent. Then, *k*_1_, *k*_2_, ⋯, *k*_*n* − *r*_ exist such that

$$\begin{array}{c} k_{1}\eta_{1}^{\prime },+k_{2}\eta_{2}^{\prime }+\cdots +k_{n-r}\eta_{n-r}^{\prime }=0 \end{array}$$ (10)

and at least one element in {*k*_1_, *k*_2_, ⋯, *k*_*n* − *r*_} is not zero. Without any loss of generality, suppose that all of the elements in {*k*_1_, *k*_2_, ⋯, *k*_*n* − *r*_} are zeros except *k*_*i*_. Then, Eq 10 can be rewritten as

$$\begin{array}{c} k_{i}\eta_{i}^{\prime }=0, \end{array}$$ (11)

i.e.,

$$\begin{array}{c} \left\{\begin{array}{ccc} & k_{i}\alpha_{i1} & =0\\ & \cdots \\ & k_{i}\alpha_{i(x-1)} & =0\\ & k_{i}\alpha_{i(x+1)} & =0\\ & \cdots \\ & k_{i}\alpha_{in} & =0\\ . \end{array}\right. \end{array}$$ (12)

Since *α*_*ix*_ = 0, then we have *k*_*i*_ *α*_*ix*_ = 0. Thus, a non-zero vector {*k*_1_, *k*_2_, ⋯, *k*_*n* − *r*_} exists such that

$$\begin{array}{c} k_{1}\eta_{1},+k_{2}\eta_{2}+\cdots +k_{n-r}\eta_{n-r}=0, \end{array}$$ (13)

which is inconsistent with the former assumption that *η* = {*η*_1_, *η*_2_, ⋯, *η*_*n* − *r*_} is a basis. Therefore, $\eta_{1}^{\prime },\eta_{2}^{\prime },\ldots ,\eta_{n-r}^{\prime }$ are mutually linear independent.

iii) Finally, we prove that the rank of the null space of **R**^**′**^ is *n* − *r*.

The rank of **R** is *r*. After removing the *x*-th column, the rank of the new matrix **R**^**′**^ is at least *r* − 1 and at most *r*. Therefore, the number of vectors in the basis for the null space of **R**^**′**^ is at least (*n* − 1) − *r* and at most (*n* − 1) − (*r* − 1). Since we have demonstrated (in ii) that there are *n* − *r* independent vectors in the null space, then the rank of the null space of **R**^**′**^ can only be *n* − *r*. Thus, the rank of **R**^**′**^ is also *r* − 1, i.e., the removal of the *x*-th column decreases the rank of the linear system by 1.

Hence, Theorem 2 is proved.

According to Theorem 2, the removal of identifiable links will not help to determine unidentifiable links. Therefore, additional identifiable links can only be obtained by the removal of unidentifiable links, so if we can determine certain unidentifiable links through *good* paths, then some other unidentifiable links will become identifiable. As mentioned earlier, this is similar to the problem of untangling a mess of kinked cables. If we cut one of the key cables, then the others can be disentangled accordingly.

### Finding the Most Important Paths

In this section, we propose a graph construction and decomposition method for finding the links that can become identifiable when a particular path is considered to be a *good* path.

#### Graph Construction

According to Theorem 2, the removal of identifiable links will not affect the attributes of other links. For a given linear system, we first remove all of the identifiable links from the matrix, before constructing an undirected graph as follows. We consider each of the links as a node; thus, if two links both appear on one path, they are neighbors of each other on the graph. For example, as shown in Table 1, *e*_1_ and *e*_4_ are identifiable links according to Theorem 1. By removing the 1-th and 4-th columns from **R**, a new matrix is obtained as follows.

$$\begin{array}{c} \bar{R}= \end{array}\left(\begin{array}{ccccc} 1 & 1 & 0 & 0 & 0\\ 0 & 0 & 0 & 0 & 0\\ 0 & 0 & 1 & 0 & 1\\ 1 & 1 & 0 & 0 & 0\\ 0 & 1 & 0 & 1 & 1\\ 0 & 0 & 1 & 0 & 1 \end{array}\right)$$ (14)

**Fig 3:**
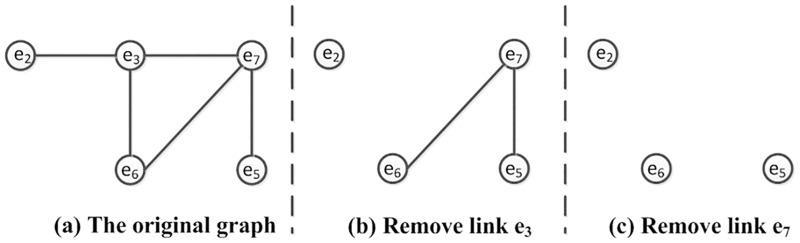
Undirected Graph from $\bar{R}$.

The undirected graph $\bar{R}$ constructed by the graph construction method is shown in Fig 3(a).

#### Graph Decomposition

In the undirected graph, the neighbors of nodes indicate the interdependencies among links. If we remove a link from the graph, the relationships between these link and their neighbors can be released. Isolated nodes in the graph denote the links that have no relationships with other links, and they can be uniquely determined in the current system.

For example, as shown in Fig 3(a), after *e*_3_ has been determined as a *good* link, we remove it from the graph. Next, *e*_2_ becomes an isolated node (as shown in Fig 3(b)), which means that it can be determined. Furthermore, by removing *e*_7_, all of the nodes in the graph are isolated and all of the corresponding links can be determined (Fig 3(c)). This also explains why path *P*_5_ (*e*_3_, *e*_6_, *e*_7_) in Table 1 is more important than the others.

Using the graph construction and decomposition method, we can find the most important path and removing it can generate the most isolated nodes.

According to Theorem 1, it should be noted that additional identifiable links can also be obtained by computing the basis of the null space of the routing matrix after removing particular rows or columns. However, the complexity of computing the basis of the null space is relatively high (in the order of *O*(*M* × *N*^2^), where *M* is the number of paths and *N* is the number of links). The additional identifiable links also need to be computed repeatedly during each round of selection. Instead, the graph decomposition method requires only *O*(*M* × P*) time, where P* is the maximum number of links over all paths (generally, P* ≪ *N*), which reduces the computational time considerably.

## Adaptive Path Selection for Loss Inference

In this section, we present our adaptive path selection algorithm (APSA) for link loss inference in network tomography applications. The algorithm comprises two steps. First, it selects the covering paths that are independent and that can cover all of the network links. Second, it sequentially selects the observed solution paths that can generate the most identifiable links.

### Selecting Covering Paths

The covering paths must be independent and they need to cover all of the links. The pseudo-code is shown in Algorithm 1, where the inputs comprise the routing matrix of all the candidate paths **R** and the measurement module **M**, while the outputs are the covering paths **P**_**C**_ and the corresponding routing matrix **R**_**C**_ of the selected paths. In each round of selection, Algorithm 1 first finds the path that can cover the most uncovered links while being independent of the selected paths (line 3). Next, it probes this path and obtains the loss rate of the path using the measurement module **M** (line 4). If the path loss rate is below 0.02, then it removes the links on this path from both the selected path matrix **R**_**C**_ and the candidate path matrix **R** (line 6 ∼ 7). Otherwise, the path is simply added to the selected matrix **R**_**C**_ (line 9). Finally, the selected path is removed from the candidate matrix **R**, the path id is added to **P**_**C**_, and the links on the selected path are removed from *L*_*uncovered*_ (line 10 ∼ 12). The algorithm continues until all of the links in the system have been covered.

**Algorithm 1**: Covering Paths Selection Algorithm

 **Input**: **R**, **M**

 **Output**: **R**_**C**_, **P**_**C**_

1 **R**_**C**_ ← ∅

2 **while** *L*_*uncovered*_ ≠ ∅ **do**

3  *p*_*max*_ = *findMaxCoverIndependentPath*(**R**)

4  *p*_*result*_ = **M**(*p*_*max*_)

5  **if** *p*_*result*_ ≤ 0.02 **then**

6   **R**_**C**_ ← *removeLinks*(**R**_**C**_, *p*_*max*_)

7   **R** ← *removeLinks*(**R**, *p*_*max*_)

8   **else**

9    **R**_**C**_ ← *addPath*(**R**_**C**_, *p*_*max*_)

10  **R** ← *removePath*(**R**, *p*_*max*_)

11  **P**_**C**_ ← *addPath*(**P**_**C**_, *p*_*max*_)

12  *L*_*uncovered*_ ← *removeCoveredLinks*(*L*_*uncovered*_, *P*_*max*_)

13 **return** **R**_**C**_, **P**_**C**_

### Selecting the Solution Paths

The solution paths are selected according to the graph construction and decomposition method. The pseudo-code for the selection algorithm is shown in Algorithm 1. The algorithm takes the candidate path matrix **R**, covering paths matrix **R**_**C**_, and measurement module **M** as inputs, and outputs the solution paths **P**_**S**_, the corresponding matrix **R**_**S**_, and the determined links **L**_**D**_. In Algorithm 1, matrix **R**_**S**_ is initialized as matrix **R**_**C**_ (line 1). During each round of selection, the algorithm first finds all of the identifiable links in the current selected matrix **R**_**S**_ and removes them from the matrix (line 3). The undirected graph **G** is constructed using **R**_**S**_ (line 4). It then selects the path from **R** that can generate the most determined links $\hat{L_{D}}$ according to the graph decomposition method and probes the path to measure the path loss rate (line 5 ∼ 6). If the path is *good*, the links $\hat{L_{D}}$ are removed from both the selected matrix **R**_**S**_ and the candidate matrix **R**, and $\hat{L_{D}}$ is added to the determined link set **L**_**D**_ (line 8 ∼ 10). Otherwise, the selected path is simply added to the selected matrix **R**_**S**_. Finally, the selected path is removed from the candidate matrix **R** and the path id is added to **P**_**S**_ (line 13 ∼ 14). The algorithm continues until no path in **R** can determine new links.

**Algorithm 2**: Solution Paths Selection Algorithm

 **Input**: **R**, **R**_**C**_, **M**

 **Output**: **R**_**S**_, **P**_**S**_, **L**_**D**_

1 **R**_**S**_ ← **R**_**C**_

2 **while** $\hat{L_{D}}\neq \emptyset$ **do**

3  [**R**_**S**_, **L**_**D**_]←*removeIdentifiableLinks*(**R**_**S**_)

4  **G** = *constructGraph*(**R**_**S**_)

5  $\left[p_{max},\hat{L_{D}}\right]=findMaxDeterminedPath\left(G,R\right)$

6  *p*_*result*_ = **M**(*p*_*max*_)

7  **if** *p*_*result*_ ≤ 0.02 **then**

8   $R_{S}\leftarrow removeLinks\left(R_{S},\hat{L_{D}}\right)$

9   $R\leftarrow removeLinks\left(R,\hat{L_{D}}\right)$

10   $L_{D}\leftarrow addLinks(L_{D}\hat{L_{D}})$

11   **else**

12    **R**_**S**_ ← *addPath*(**R**_**S**_, *p*_*max*_)

13  **R** ← *removePath*(**R**, *p*_*max*_)

14  **P**_**S**_ ← *addPath*(**P**_**S**_, *p*_*max*_)

15 **return** **R**_**S**_, **P**_**S**_, **L**_**D**_

## Evaluation

In the evaluation, we compared our algorithm (APSA) with a state-of-the-art approach (SelectPath) based on extensive simulations.

### Evaluation Setup

Topologies: We conducted our experiments using the realistic ISP topologies from the Rocketfuel Project [19]. We selected the *AS*1239 and *AS*3356 topologies with relatively large scales, as well as *AS*1755 and *AS*6461 with relatively small scales to evaluate the performance of the algorithms at different network scales. The numbers of nodes and links in each AS topology are presented in Table 2.

**Table 2:** Details of the Topologies.

Topologies	AS1239		AS1755		AS3356		AS6461	
Nodes	604		172		624		182	
Links	4536		762		10596		588	
Monitors	40	60	40	60	40	60	40	60
Candidate paths	1560	3540	1560	3540	1560	3540	1560	3540
Covered Links	524	740	221	273	787	1261	147	181

Candidate Paths: We randomly selected 40 and 60 nodes as the monitors that could be both initiating and receive probes. The candidate paths were generated between each monitor pair and we adopted a shortest path routing policy for all of the topologies in Table 2. Links that could not be covered by any paths were removed from the system. The numbers of candidate paths and the links covered by these paths are listed in Table 2.

Link Loss: We allowed each link to be congested with a probability *p*. This probability affected the selection result in our experiments, so we varied the value of *p* to evaluate the performance of the two algorithms. As the loss model could affect the performance of our algorithm, we used three different packet loss models in the experiments as follows.

**Model 1**: This model was proposed by [20] and is also used in [14] and [21]. In this model, congested links had loss rates with a uniform distribution in [0.05, 0.2] and good links had loss rates in [0, 0.002]. The lossy links are selected randomly from all network links.

**Model 2**: In this model, the lossy links in the underlying network are also selected randomly and the link loss rates are drawn from a lognormal distribution with mean 0.04 and standard deviation 0.1^6^. The loss rates of the remaining links are set to zero. This model is used in [22], where the parameters are estimated from a large set of loss rate measurements in about 3,600 Planetlab paths.

**Model 3**: As Ghita et. in [13] show with their experimental data, more than half of the lossy links in each round are edge links and congestion mostly happens close to the end-hosts. In this model, we choose lossy links based on a “weight” parameter: links that are closer to the end-hosts (i.e., monitors that send and receive packets) are assigned a larger “weight.” Congested links also have loss rates with a uniform distribution in [0.05, 0.2] and good links have loss rates in [0, 0.002].

After assigning each link a loss rate, the actual losses on each link followed a Gilbert process. In the Gilbert model, a link fluctuates between good and congested states. The links did not drop any packets when in the good state, whereas they dropped all of the packets when in a congested state.

### Baselines and Metrics

We compared our new algorithm (APSA) with the state-of-the-art path selection approach for network tomography called SelectPath [2]. There are also several methods that measure the whole candidate path set to infer the link loss rates without path selection [12][13][14]. Thus, we also present the performance using all of the candidate paths given their measurement results in our figures (denoted as “All Paths”).

The performance of each approach was evaluated based on the following three metrics. i) Cost. The number of paths selected. ii) Quality. The number of links that could be determined accurately from the selected paths given the loss rates on these paths. iii) Computational Time. The time period between when the routing matrix was input and the selected paths were returned.

All of the figures show the statistical results based on 20 runs.

### Evaluation Results

#### Number of Paths Selected

**Fig 4:**
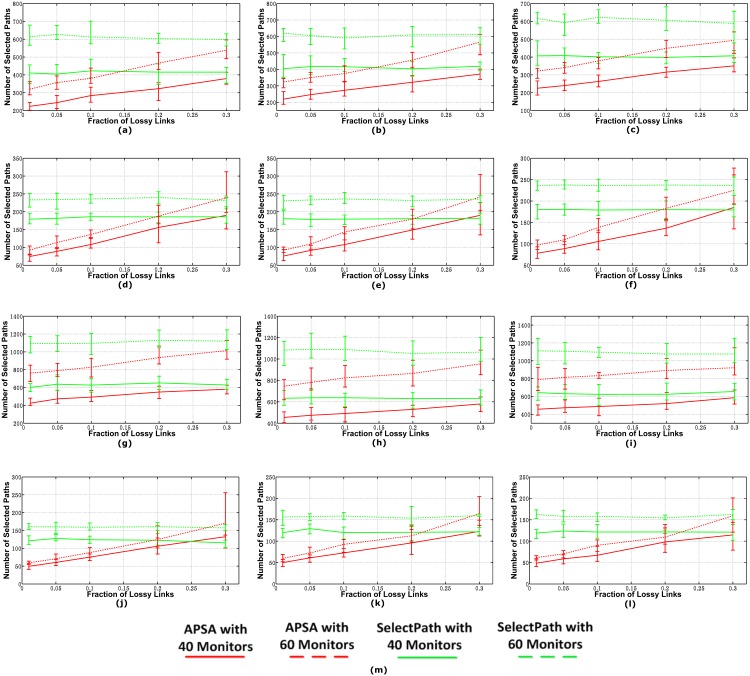
Number of Paths Selected by the Two Algorithms. (a)AS1239 in model 1(b)AS1239 in model 2(c)AS1239 in model 3(d)AS1755 in model 1(e)AS1755 in model 2(f)AS1755 in model 3(g)AS3356 in model 1(h)AS3356 in model 2(i)AS3356 in model 3(j)AS6461 in model 1(k)AS6461 in model 2(l)AS6461 in model 3(m)Legend.

In Fig 4, we plot the mean, maximum, and minimum number of paths selected by the two algorithms because we varied the fraction of lossy links in the topologies. The paths selected by APSA included both covering paths and solving paths. According to Fig 4, APSA selected far fewer paths than the SelectPath algorithm for all three models, especially for the networks with relatively less lossy links. This advantage was even more obvious when there were more monitors in the networks. In the networks with a relatively small number of lossy links, APSA usually presented a relatively stable performance (i.e., the sample deviation is relatively small), but the deviations increased when the fraction of lossy links increased. Fewer lossy links resulted in more *good* paths, so the number of paths selected by ASAP increased linearly with the fraction of lossy links. However, for most of the communication networks, the fraction of lossy links was generally less than 15%. Thus, compared with SelectPath, ASAP reduced the probing cost by more than 50% in the networks with less than 15% lossy links.

#### Number of Links Determined

**Fig 5:**
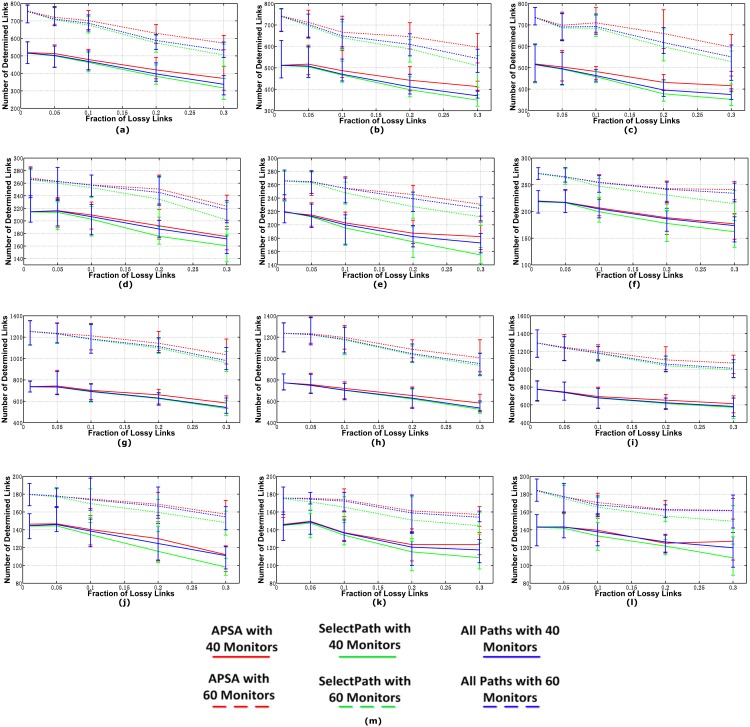
Number of Links that could be Determined Accurately in the Three Scenarios. (a)AS1239 in model 1(b)AS1239 in model 2(c)AS1239 in model 3(d)AS1755 in model 1(e)AS1755 in model 2(f)AS1755 in model 3(g)AS3356 in model 1(h)AS3356 in model 2(i)AS3356 in model 3(j)AS6461 in model 1(k)AS6461 in model 2(l)AS6461 in model 3(m)Legend.

The numbers of links that could be determined accurately according to the loss rates on all of the candidate paths and the paths selected by APSA and SelectPath, respectively, are shown in Fig 5. In all three models, the curve obtained for APSA was the highest of the three curves under all of the different topologies and different models. Therefore, APSA could use the least number of paths to determine the most links. For this metric, The samples for the three curves present similar deviations under different numbers of lossy links, which indicates that the fraction of lossy links does not affect the stability of either APSA or SelectPath. In all three scenarios, we considered the links determined by the *good* paths. Thus, all three curves declined as the fraction of lossy links increased. We also note that the gap between the curves widens when the fraction of lossy links increases. This is because when the fraction of lossy links is low, most paths will present *good* results. Because all three scenarios remove *good* links on *good* paths before they perform the inference, these *good* links makes up the vast majority of their determined links. As a result, the advantage of APSA seems less apparent. As the fraction of lossy links grows, APSA is clearly superior. We will show in the “Inapplicable Cases” that the gap between the curves narrows when the fraction increases to a certain value.

The paths selected by APSA could determine even more links than all of the candidate path set because APSA gradually removes links that can be determined by the current path loss rates, including both *good* links and lossy links that are currently identifiable according to the graph decomposition method. After the lossy links have been removed, some of the lossy paths in the current round become *good*, thereby leading to more determined links.

#### Running Speed

**Fig 6:**
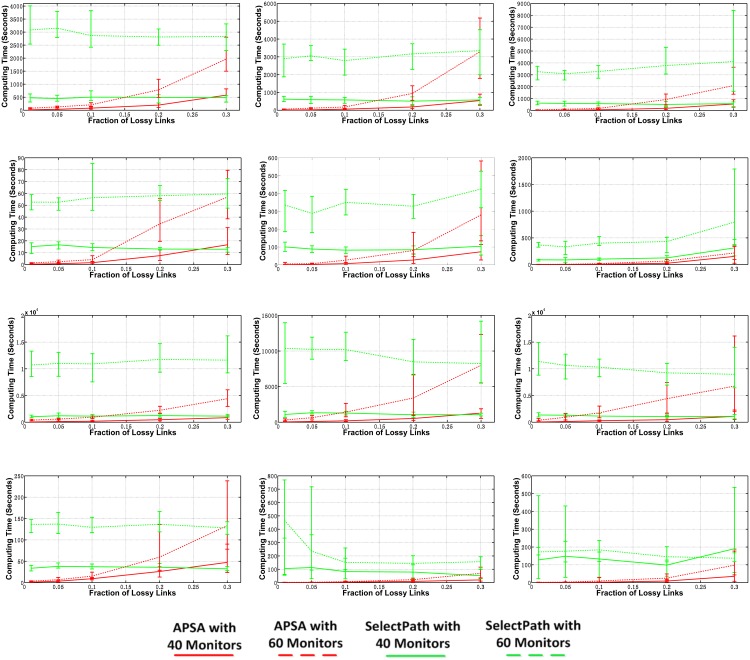
Computational Times for the Two Algorithms. (a)AS1239 in model 1(b)AS1239 in model 2(c)AS1239 in model 3(d)AS1755 in model 1(e)AS1755 in model 2(f)AS1755 in model 3(g)AS3356 in model 1(h)AS3356 in model 2(i)AS3356 in model 3(j)AS6461 in model 1(k)AS6461 in model 2(l)AS6461 in model 3(m)Legend.

We plot the computational times of the two algorithms in Fig 6, which shows that APSA was much faster than SelectPath in most cases. APSA removes the links that can be determined in each selection round according to the current path loss rates, and thus the scale of the system is then reduced round by round. As the fraction of the lossy links increases, fewer links can be determined from the *good* paths. Thus, the computational time of our algorithm increases accordingly. We can also learn from the figure that, for both 40-monitor and 60-monitor cases, the samples’ APSA deviations are extremely small when the fraction of lossy links is under 10% and the deviations become relatively large when the lossy fraction grows to 20%. Furthermore, for SelectPath, the samples are relatively stable in the 40-monitor networks, but fluctuate drastically in the 60-monitor networks, which cover many more network links than the 40-monitor networks. This indicates that the fraction of lossy links has a relatively large influence on the computing time of APSA, while the scale of the network affects SelectPath more. Furthermore, the gap between the two curves widens even further in the networks with 60 monitors, which means that APSA is more appropriate for large networks with relatively fewer lossy links.

#### Inapplicable Cases

**Fig 7:**
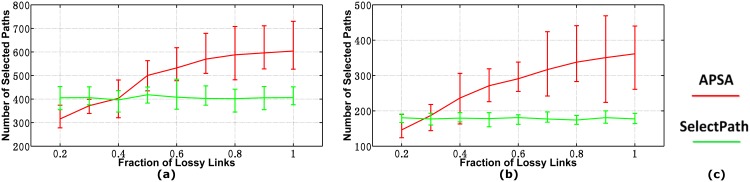
Number of Selected Paths in Inapplicable Cases. (a)AS1239(b)AS1755(c)Legend.

In above figures, we know that APSA will perform much better than SelectPath when the fraction of lossy links is relatively small. However, we also would like to explore the performance of our algorithm in cases where our algorithm cannot hold. We allow the fraction of lossy links to vary from 20% to 100%, and plot the performance of the two algorithms for all metrics. The percentages of *good* paths under different fractionsof links are shown in Table 3. Because there is little difference between the 40-monitor cases and 60-monitor cases and the curves for AS3356 and AS6461 are similar to those of AS1239 and AS1755, respectively, we only show our results on AS1239 and AS1755 in the 40-monitor networks. In Fig 7, we can see the intersections at 39% and 28%, respectively, which indicate that if the fraction of lossy links is lower than 39% (for a relatively large network) and 28% (for a relatively small network), APSA will select fewer paths than SelectPath. Otherwise, APSA requires more paths than SelectPath.

**Table 3:** The Percentages of *Good* Paths.

Fraction of lossy links	Percentage of *good* paths	
	AS1239	AS1755
0.2	0.3956	0.3443
0.3	0.2403	0.2239
0.4	0.1513	0.1200
0.5	0.0738	0.0702
0.6	0.0430	0.0340
0.7	0.0176	0.0224
0.8	0.0049	0.0094
0.9	0.0035	0.0040
1.0	0	0

**Fig 8:**
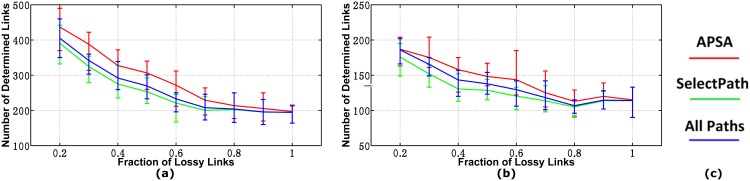
Number of Determined Links in Inapplicable Cases. (a)AS1239(b)AS1755(c)Legend.

We plot the number links that can be accurately determined in Fig 8. In this figure, the gap between APSA and SelectPath increases until the fraction goes up to 60%, and then reduces gradually. This is because when the fraction of lossy links exceeds 60%, very few paths (less than 5%) will present *good* results, and the help from *good* links becomes trivial. However, APSA always determines the most links of all three scenarios for all plotted values.

**Fig 9:**
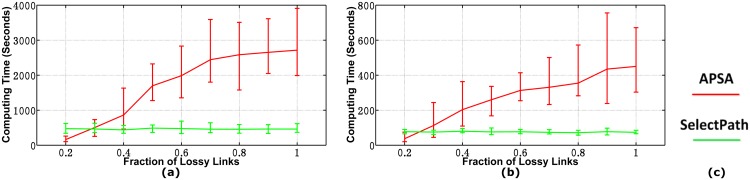
Computational Times in Inapplicable Cases. (a)AS1239(b)AS1755(c)Legend.

Fig 9 shows the computational times of the two algorithms when the fraction of lossy links varies from 0.2 to 1. In this figure, the two curves intersect at 30% and 27%, respectively. Furthermore, the curves of APSA increase linearly with the fraction of lossy links while the curves of SelectPath keep flat for the entire range of tested values.

#### Summary of the results

APSA is quite suitable for networks with the lossy fraction that is under 30%. It requires notably fewer measurement paths and less computational time than the baseline to determine many more network links. This superiority becomes even more prominent in relatively large networks. When the fraction of lossy links exceeds 30%, the performance of APSA drops. It requires more measurement paths and computational time than the baseline. Moreover, the number of selected paths and computational time grows linearly with the fraction of lossy links. However, the number of links that can be determined by APSA is still larger as the fraction of lossy links varies from 1% to 100%. Nevertheless, a substantial number of studies [13][22][23][14] have demonstrated that the loss of packets is not quite so extensive in general networks. [13] pointed out that in their experimental data based on Plantlab, about 83% of links had a negligible loss rate, while only 4% had a loss rate above 0.05. Besides, all current studies on network loss rates (such as [2][13][14] and [23]) as far as we know, similarly allow the fraction of lossy links to vary from 0 to 30% when they set up their simulation environments.

## Conclusion

In this study, we proposed an adaptive path selection method for network tomography applications based on link loss inference. In our proposed method, the probing paths are selected round by round based on the loss rates of paths that have been selected previously. We also proposed Theorem 2 as well as a graph construction and decomposition method to find the path that can provide the maximum information to determine the current system during each round of selection. According to extensive simulations based on realistic ISP topologies, our results showed that the proposed method (APSA) required much lower probing costs and a shorter run time to accurately determine more network links compared with the classical SelectPath approach.

Selecting paths adaptively can make use of the information from *good* paths, as well as performing well in the presence of network element failures. If the loss rate of a selected path is 1, then one or more links on the path is in a faulty state. In this case, we should first select the paths that overlap with the failure path to locate the failed links. In addition, selecting paths that do not include the failure links can avoid more failed paths. These improvements will be made in our future research.

## Supporting Information

S1 File Algorithms’ Source Codes. (ZIP)
